# The effectiveness of Tai Chi in improving depressive mood among young individuals aged 15–24 years: a systematic review and meta-analysis

**DOI:** 10.3389/fpubh.2025.1517350

**Published:** 2025-09-03

**Authors:** Bozhen Huang, Meijiao Zhou, Min Jiang, Shanshan Song, Lei Liu, Luyao Wang, Liang Wang, Yanhong Jian, Ping Wang, Tao Yang, Xinjue Shan, Xinqian Liu

**Affiliations:** ^1^Beijing Shijitan Hospital, Capital Medical University, Beijing, China; ^2^First Teaching Hospital of Tianjin University of Traditional Chinese Medicine, Tianjin, China; ^3^School of Medical Humanities, Capital Medical University, Beijing, China; ^4^Beijing University of Chinese Medicine, Beijing, China

**Keywords:** Tai Chi, depressive mood, young individuals, mental health, exercise, meta-analysis

## Abstract

**Background:**

Depression is a leading mental health concern among young individuals aged 15–24 years. Tai Chi, a low-cost, low-risk practice that has been shown to significantly alleviate depressive mood in the middle-aged and older adults, has not been rigorously evaluated in young individuals aged 15–24 years.

**Methods:**

RCTs evaluating Tai Chi therapy for young individuals aged 15–24 years with depressive mood were retrieved from several databases, including PubMed, Embase, Web of Science, Cochrane Library, CNKI, Wanfang Database, VIP Database, and CBM, covering the period from the database inception to May 18, 2025. The Cochrane Risk of Bias tool was employed to assess the bias risk in all included studies. Following a thorough screening process, data extraction, and coding, a meta-analysis was conducted using RevMan 5.2 and Stata 12.0.

**Results:**

A total of 11 articles were included in the analysis, comprising 782 participants, with 434 in the Tai Chi intervention group and 348 in the control group. The meta-analysis results indicated that Tai Chi therapy effectively reduced depression scale scores in young individuals aged 15–24 years with depressive mood compared to the control group [SMD = −0.80, 95% CI (−1.14, −0.46), *p* < 0.001], but heterogeneity was high (*I*^2^ = 79.3%). Subgroup analyses showed that the intervention effect was optimal when the duration was 12 weeks [SMD = −1.11, 95% CI (−1.77, −0.45), *p* < 0.001]. The most significant intervention effect was observed when participants practiced Tai Chi for >3 h per week [SMD = −2.03, 95% CI (−3.63, −0.43), *p* < 0.001]. Sensitivity analyses indicated that the results of this study were robust and reliable. Publication bias was indicated by funnel plot asymmetry and Egger’s test (*p* = 0.042). The trim-and-fill analysis adjusted the pooled SMD from −0.80 (*I*^2^ = 79.30%) to −0.94 (*I*^2^ = 83.80%), indicating that the initial conclusion remained robust even in the presence of possible publication bias.

**Conclusion:**

As a non-pharmacological intervention, Tai Chi shows great promise in addressing depressive mood in young individuals aged 15–24 years within the field of mental health, warranting further research and promotion.

**Systematic review registration:**

https://www.crd.york.ac.uk/PROSPERO/, identifier: CRD42024580026.

## Introduction

Depression is a severe mental disorder. Data from the Global Burden of Disease Study 2019 indicate an increasing burden of depression among young patients aged 10–24 years, with the global number of prevalent cases rising by 21.67% between 1990 and 2019. During this period, age-standardized prevalence rate increased in high-income regions such as North America and Australasia, while relative declines were observed in regions like South Asia and East Asia (1). This rise is particularly concerning among youth defined by the World Health Organization (WHO) as aged 15–24 years, a pivotal transition from dependence to independence marked by academic, employment, and financial pressures. These stressors significantly heighten the risk of developing depressive disorders in this population (2). The etiology of depression is complex, primarily influenced by genetic predisposition, metabolic disturbances, and life events (3). Current treatment options for depression mainly include pharmacotherapy (4) and psychotherapy (5). However, the former can lead to dependency and have adverse effects (6) and the latter requires continuous support from a mental health professional and is costly and less convenient. Therefore, there is an urgent need for a treatment that is effective, has minimal side effects, and is easily implementable (7).

A growing body of evidence indicates that non-pharmacological interventions, including yoga and meditation, demonstrate efficacy in ameliorating depressive mood (8–10). Yoga involves static postures and often requires mats or props, which can increase joint pressure. Tai Chi uses slow, low-impact movements that are joint-friendly and do not require equipment, making it more accessible for young individuals aged 15–24 years. Compared with meditation, Tai Chi’s movement helps enhances engagement. Its holistic nature may alleviate depressive symptoms through several mechanisms, including modulation of the autonomic nervous system, reduction of neuroinflammation, and enhancement of neuroplasticity (11, 12). Evidence suggests that specific styles, such as the 24-style Tai Chi, and extended practice durations yield more pronounced improvements in depressive mood (13). These findings underscore Tai Chi’s potential as an effective intervention for depression.

Previous clinical trials (14, 15) have shown that Tai Chi can alleviate depressive symptoms among middle-aged and older adults. However, the efficacy of Tai Chi for treating depressive mood in young individuals aged 15–24 years remains controversial (16, 17). Therefore, this study conducted a systematic review and meta-analysis to evaluate the therapeutic effects of Tai Chi on depressive mood in young individuals aged 15–24 years. Moreover, we provide evidence-based recommendations on optimal intervention period and weekly training time for Tai Chi therapy in this age group, aiming to guide clinicians in practice.

## Methods

This systematic review and meta-analysis followed the Preferred Reporting Items for Systematic Reviews and Meta-Analysis (PRISMA, 2020) guidelines (18). The protocol is registered with PROSPERO (CRD42024580026).

### Literature search

A comprehensive electronic search was conducted across PubMed, Embase, Web of Science, Cochrane Library, China National Knowledge Infrastructure (CNKI), Wanfang (WF) Database, VIP Database (VIP), and Chinese Biomedical Literature Database (CBM). The search strategy was a combination of subject term and free text searches, using keywords such as (“Tai Chi” OR “Tai Ji” OR “Tai Chi Chuan”) and (“Depressive Disorder” OR “Depression” OR “Depressive Symptoms”) and (“randomized controlled trial” OR “random” OR “random*” OR “placebo”), integrated through Boolean logic. All relevant literature from the inception of each database up to May 18, 2025 were retrieved. The complete search strategy for each database is detailed in Supplementary Table 1. Because we specifically focused on trials enrolling participants aged 15 to 24 years, the pool of eligible randomized controlled trials (RCTs) was inherently limited, resulting in a relatively small number of studies for inclusion.

### Inclusion and exclusion criteria

Inclusion criteria were: (1) young individuals aged 15–24 years with depressive mood. All included participants exhibited either clinically diagnosed depression or subclinical depressive symptoms; (2) interventions included various types of Tai Chi (e.g., 24-style Tai Chi, Chen-style Tai Chi, Yang-style Tai Chi); (3) participants in the control group were instructed to maintain their usual lifestyle and avoid any specific interventions such as Tai Chi, yoga, or meditation; (4) at least one type of outcome scale related to depressive symptoms included; (5) RCTs.

Exclusion criteria were: (1) participants receiving antidepressant drugs, herbal remedies, psychotherapy or any other adjunctive treatments during the study period; (2) depressive state secondary to other medical conditions; (3) duplicate publications; (4) significant quality problems, including: inappropriate choice of statistical test, miscalculation or misreporting of *p*-values, inconsistent reporting of effects, obvious data typos or impossible numbers; (5) incomplete descriptions of the resulting data, particularly when authors fail to respond to requests for clarification; (6) systematic reviews, meta-analyses, reviews; (7) non-peer-reviewed papers, such as conference papers, newspapers, etc.

### Literature screening process

The collected literature was imported into EndNote 21 according to the research strategy, and duplicate articles within the database were excluded. Two researchers conducted a preliminary screening of the titles and abstracts based on predetermined inclusion and exclusion criteria, followed by a detailed full-text review for further selection. The researchers cross-verified their respective screening results. If their conclusions were consistent, the articles were included in the study. In cases of disagreement, a third researcher was consulted until a consensus was reached. Information was extracted from the final included articles, and the risk of bias was recorded using a predetermined method.

### Data extraction

Data extraction consists of: (1) basic information on research: title, first author, year of publication, type of research; (2) subject characteristics: country, sample size, gender; (3) the intervention group: type of exercise, intervention period, weekly training time, exercise intensity, outcome; (4) the control group: outcome; (5) information related to the assessment of the quality of literature.

### Risk of bias evaluation of the included literature

In accordance with the Cochrane Collaboration guidelines (19), two researchers independently assessed the quality of the included literature using the tools recommended in the Cochrane version 5.1 manual. The assessment consisted of seven levels: (1) random sequence generation (selection bias); (2) allocation concealment (selection bias); (3) blinding of participants and personnel (performance bias); (4) blinding of outcome assessment (detection bias); (5) incomplete outcome data (attrition bias); (6) selective reporting (reporting bias); (7) other bias. It assessed each study in terms of three dimensions: high risk of bias, unknown risk of bias, and low risk of bias. Any inconsistencies in the assessments were resolved through discussions with a third researcher.

### Statistical analysis

Analyses employed RevMan 5.2 and Stata 12.0. Continuous outcomes were pooled as standardized mean differences (SMDs) with 95% CIs. Heterogeneity was quantified using Cochran’s *Q* test (significance threshold *p* ≤ 0.10) and *I*^2^ statistics (20). For all 11 included studies, the random-effects model was applied to address inherent heterogeneity. When *I*^2^ < 25%, random-effects estimates were observed to closely approximate fixed-effect results. Two-sided tests employed *α* = 0.05 significance threshold. Publication bias was assessed through funnel plot symmetry, Egger’s test (21), and Begg’s test (22). Observed asymmetry prompted trim-and-fill adjustment for potential missing studies (23).

## Results

### Literature search and screening

**Figure 1:**
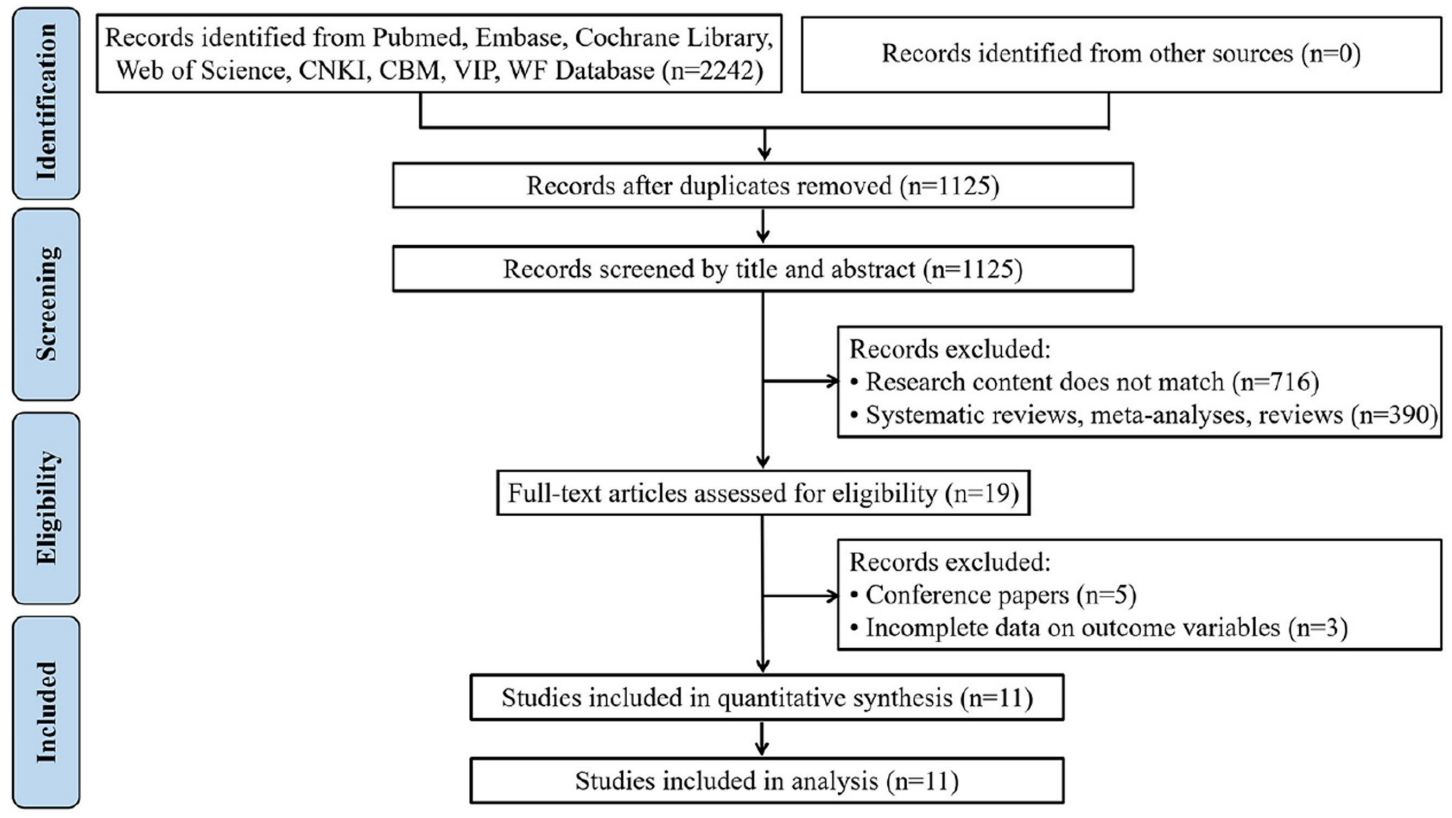
Flow diagram of the selection process of literature.

A total of 2,242 documents were obtained during the initial screening. Non-compliant documents were systematically eliminated through a careful review of weights, titles and full texts. Ultimately, 11 studies were included in the final analysis, as shown in Figure 1.

### Basic characteristics of the included literature

A total of 11 RCTs were included, all published in Chinese or English, and spanning the period 2003 to 2024. Type of intervention is mainly 24-style Tai Chi. Intervention period ranges from 8 to 18 weeks. Weekly training time ranges from 80 to 400 min. The exercise intensity of the intervention group ranged from very light to moderate. As shown in Table 1.

**Table 1:** Basic characteristics of the literature included.

References	Country	Sample size (T/C)	Gender (M/F)	Type of intervention	Intervention period	Weekly training time	Exercise intensity	Assessment tools
Chen et al. (2019) (38)	China	18/18	0/36	24-style Tai Chi	16 weeks	180–240 min/week	Moderate	CES-D
Gao et al. (2012) (39)	China	60/30	46/44	Tai Chi	16 weeks	100 min/week	Moderate to Light	SCL-90
Hua and Sun (2021) (40)	China	60/20	31/49	24-style Tai Chi	12 weeks	80 min/week	NR	DASS
Li and Yin (2008) (41)	China	20/18	0/38	24-style Tai Chi	8 weeks	180 min/week	Light to Very Light	POMS
Mao et al. (2008) (24)	China	52/52	0/104	24-style Tai Chi	18 weeks	180 min/week	Moderate	SDS
Su (2010) (25)	China	30/15	0/45	24-style Tai Chi	12 weeks	160 or 400 min/week	Moderate to Light	BDI
Wang (2006) (42)	China	23/18	NR	24-style Tai Chi	12 weeks	190 min/week	NR	POMS
Wu et al. (2023) (26)	China	49/54	33/70	24-style Tai Chi	12 weeks	180 min/week	NR	PHQ-9
Xie (2020) (27)	China	31/32	15/48	24-style Tai Chi	12 weeks	180 min/week	Moderate	PHQ-9
Yang (2003) (43)	China	51/51	NR	24-style Tai Chi	8 weeks	180 min/week	NR	SCL-90
Zou and Li (2024) (44)	China	40/40	NR	24-style Tai Chi	16 weeks	90 min/week	NR	SCL-90

T, treatment group; C, control group; M, male; F, female; NR, not reported; CES-D, center for epidemiologic studies depression scale; SCL-90, symptom checklist 90; DASS, depression, anxiety, and stress scale; POMS, profile mood states; SDS, self-rating depression scale; BDI, beck depression inventory; PHQ-9, patient health questionnaire-9.

### Quality assessment and risk of bias

**Figure 2:**
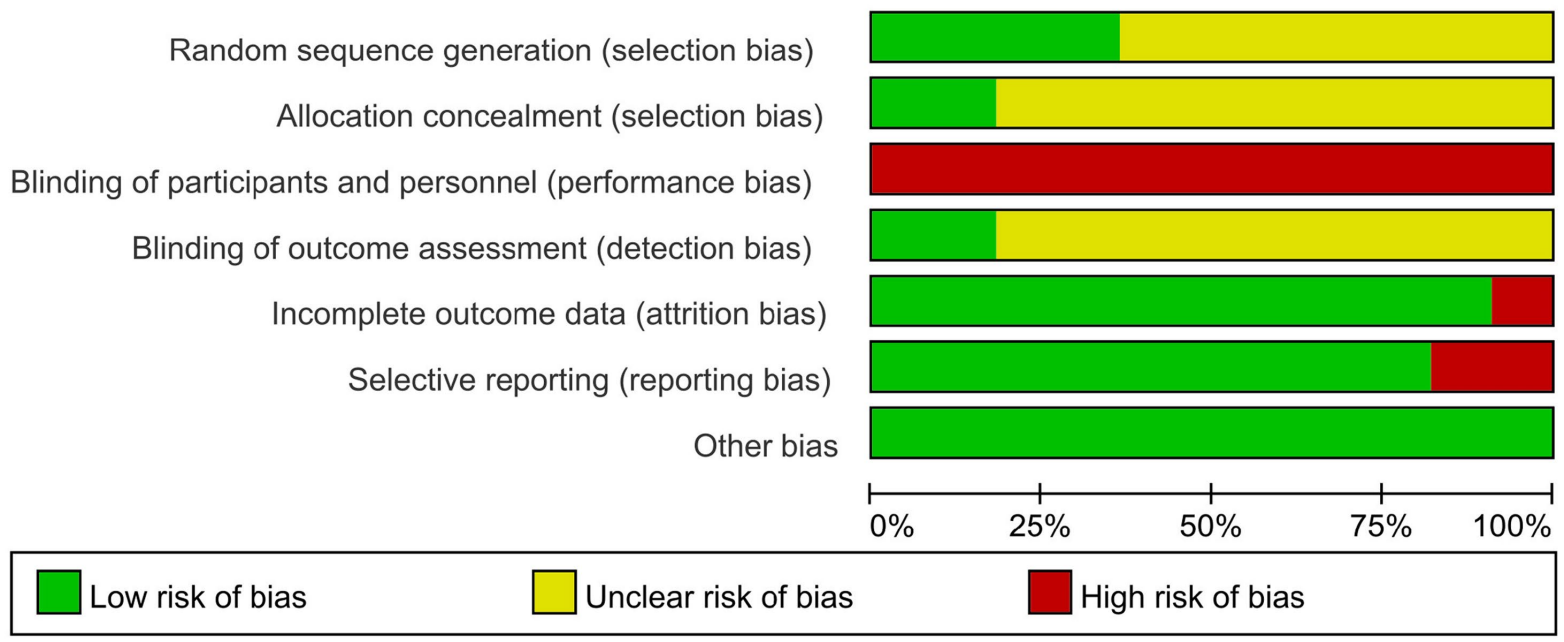
Risk of bias graph for all included studies.

**Figure 3:**
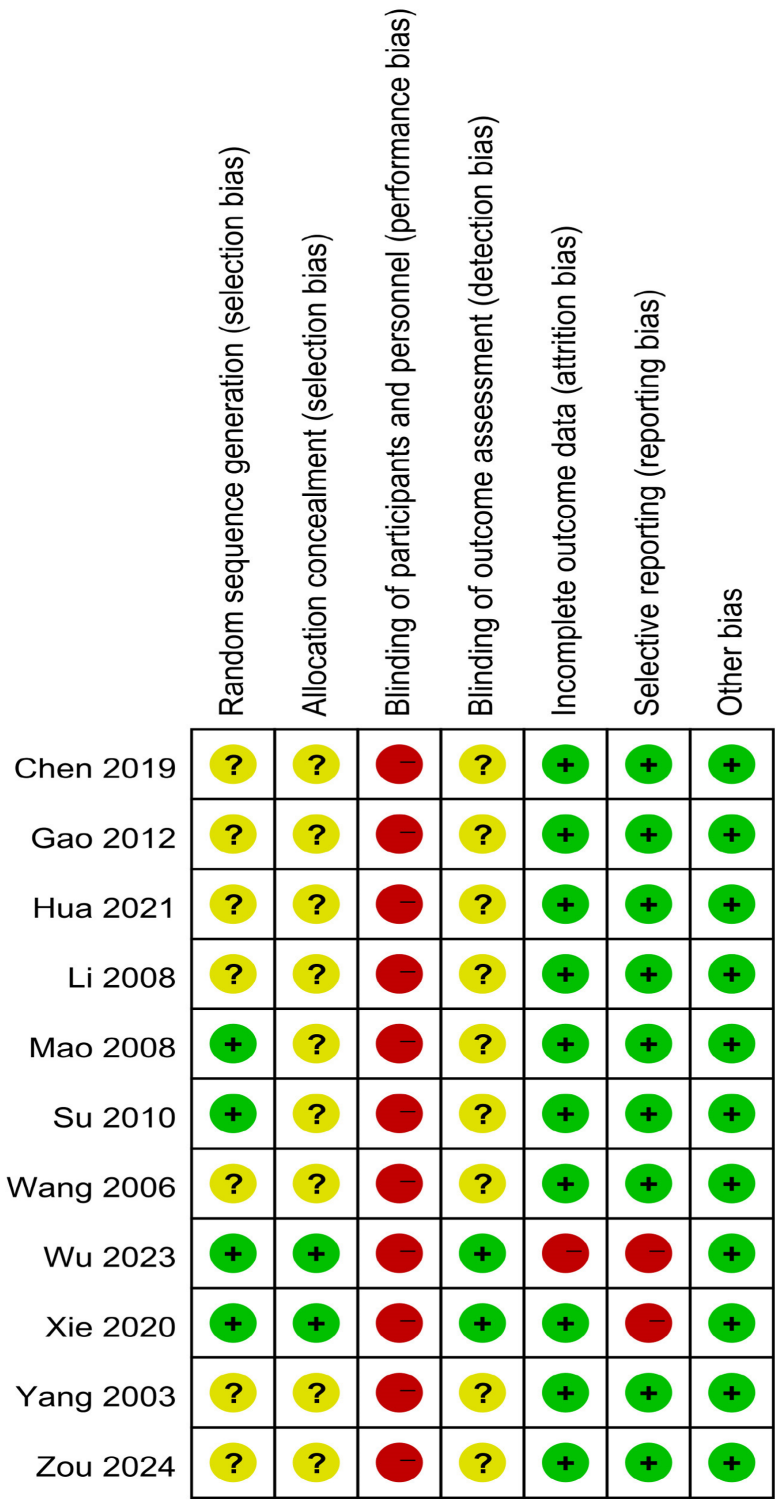
Risk of bias summary for all included studies.

Four studies (24–27) clearly detailed their randomization methods, such as computer-generated random numbers. Two studies (26, 27) outlined their allocation concealment methods, such as the sealed envelope technique. Due to the nature of the exercise intervention, blinding was not feasible for either the researchers or the participants. Two studies (26, 27) noted that the statistical analysts were not involved in the trial’s delivery or data collection. In one study (26), the intervention group had a high rate of loss of follow-up, and there was attrition bias. Two studies (26, 27) selectively reported outcomes. No additional source of biases were identified in the reviewed literature, as shown in Figures 2, 3. The basis for the risk of bias assessment is detailed in Supplementary Table 2.

### Meta-analysis of depression scale scores

A total of 11 articles, comprising 782 participants were included in the analysis, with 434 individuals in the intervention group and 348 in the control group. The results revealed no statistically significant difference in depressive symptom scores between the intervention group and the control group before treatment (*p* > 0.05), demonstrating comparability between the two groups, as shown in the Supplementary Figure 1.

**Figure 4:**
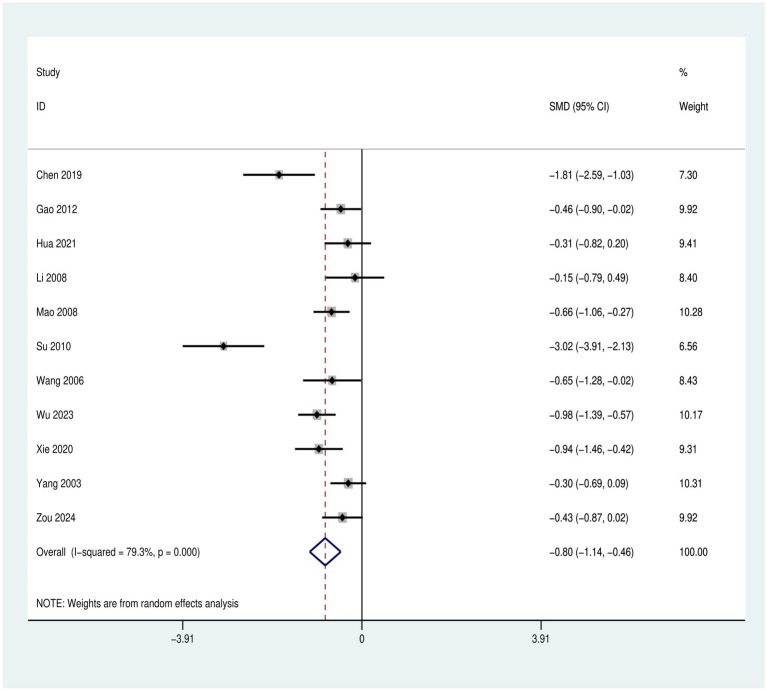
Forest plot of Tai Chi’s effects on depressive mood in young individuals aged 15–24 years.

The post-intervention analysis indicated that, the depression scale scores in the intervention group were significantly lower than those in the control group [SMD = −0.80, 95% CI (−1.14, −0.46), *p* < 0.001], suggesting that Tai Chi is effective in improving depressive mood among young individuals aged 15–24 years, as shown in Figure 4.

### Meta-regression analysis of heterogeneity

To explore potential sources of heterogeneity, we conducted a meta-regression analysis examining six covariates: (1) Gender distribution; (2) Type of Tai Chi intervention; (3) Intervention period; (4) Weekly training time; (5) Exercise intensity; (6) Depression assessment tools. The results indicated no statistically significant moderators (all *p* > 0.05). Substantial residual heterogeneity persisted (*I*^2^_res_ = 84.12%), with minimal between-study variance explained by the model (Adj *R*^2^ = −22.63%). The joint test for all covariates was non-significant [*F*(6,4) = 0.92, *p* = 0.5579, Knapp–Hartung adjustment], as shown in Table 2.

**Table 2:** Meta-regression analysis of heterogeneity sources.

Covariate	Coefficient	Std. Err.	*t*-value	*P*-value	95% CI
Gender distribution	−1.02	1.025	−0.99	0.376	−3.87, 1.83
Intervention type	0.918	1.513	0.61	0.577	−3.28, 5.12
Intervention period	0.589	0.719	0.82	0.459	−1.41, 2.59
Weekly training time	0.458	0.89	0.51	0.634	−2.01, 2.93
Exercise intensity	−0.534	1.046	−0.51	0.636	−3.44, 2.37
Assessment tools	−1.266	0.73	−1.73	0.158	−3.29, 0.76
Intercept	−0.194	2.52	−0.08	0.942	−7.19, 6.80

*τ*^2^ = 0.5586; *I*^2^_res_ = 84.12%; Adjusted *R*^2^ = −22.63%.

### Subgroup analysis

#### Intervention period

In the assessment of depressive symptoms using the 11 articles, the difference in depressive symptom scores between the experimental and control groups in the 3 subgroups prior to treatment was not statistically significant (*p* > 0.05), as shown in the Supplementary Figure 2.

After treatment, when the intervention period was ≥6 weeks and <12 weeks, the intervention effect in the experimental group was [SMD = −0.26, 95% CI (−0.59, 0.08), *p* = 0.701]. When the intervention duration was 12 weeks, the intervention effect in the experimental group was [SMD = −1.11, 95% CI (−1.77, −0.45), *p* < 0.001]. When the intervention duration was >12 weeks and ≤18 weeks, the intervention effect in the experimental group was [SMD = −0.74, 95% CI (−1.19, −0.29), *p* = 0.018].

**Figure 5:**
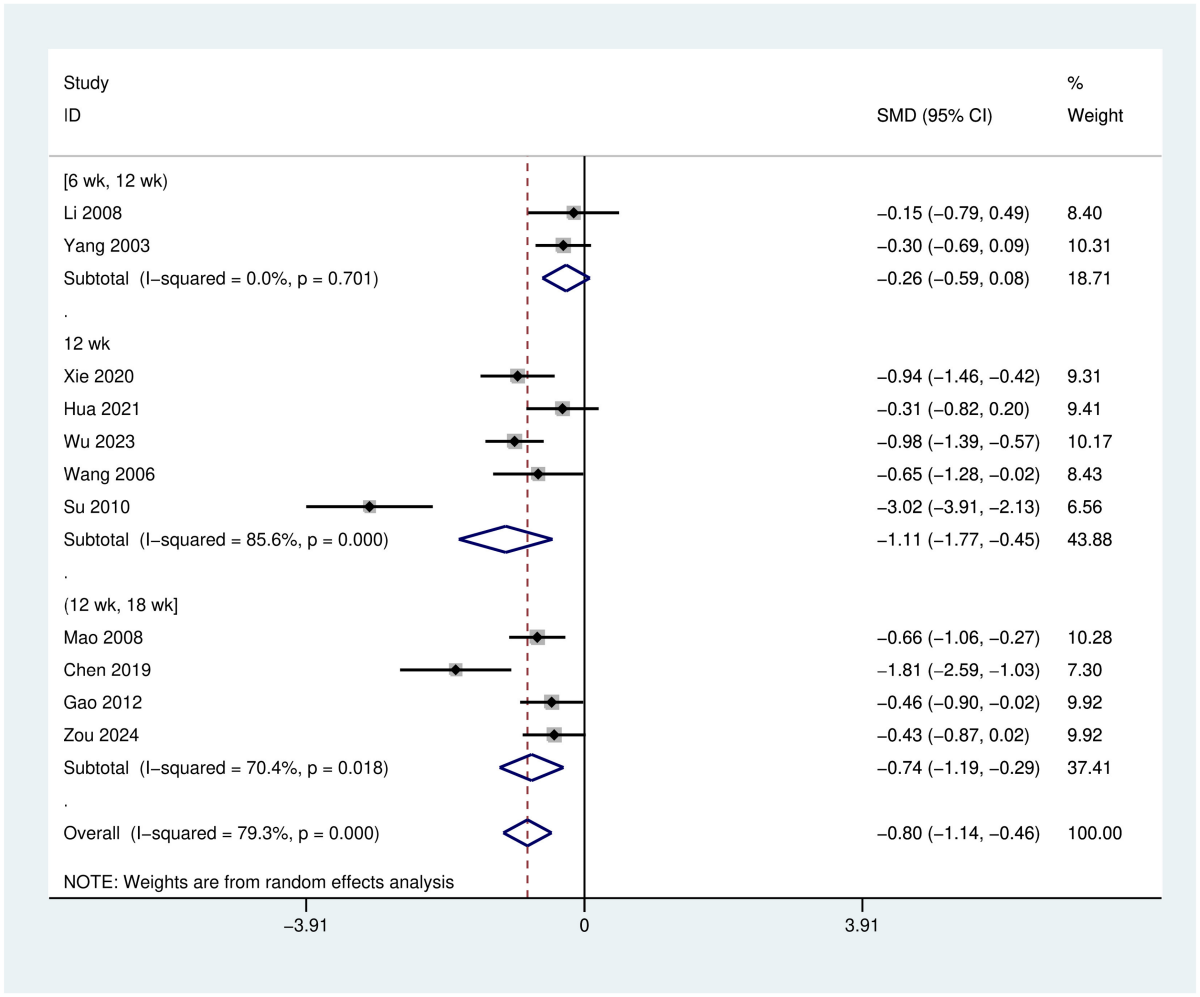
Forest plot for subgroup analysis of Tai Chi intervention period.

Between-group comparisons revealed that the intervention effect was optimal when the duration was 12 weeks. In contrast, the intervention effect was poorest when the duration was ≥6 weeks and <12 weeks. The effect was intermediate when the intervention duration was >12 weeks and ≤18 weeks, as shown in the Figure 5.

#### Weekly training time

Before treatment, there was no statistically significant difference in depressive symptoms scores between the experimental and control groups (*p* > 0.05), as shown in the Supplementary Figure 3.

After treatment, when weekly training time was ≥1 h and ≤2 h, the intervention effect in the experimental group was [SMD = −0.41, 95% CI (−0.67, −0.14), *p* = 0.902]. When weekly training time was >2 h and ≤3 h, the intervention effect in the experimental group was [SMD = −0.75, 95% CI (−1.13, −0.38), *p* = 0.005]. When weekly training time was >3 h, the intervention effect in the experimental group was [SMD = −2.03, 95% CI (−3.63, −0.43), *p* < 0.001].

**Figure 6:**
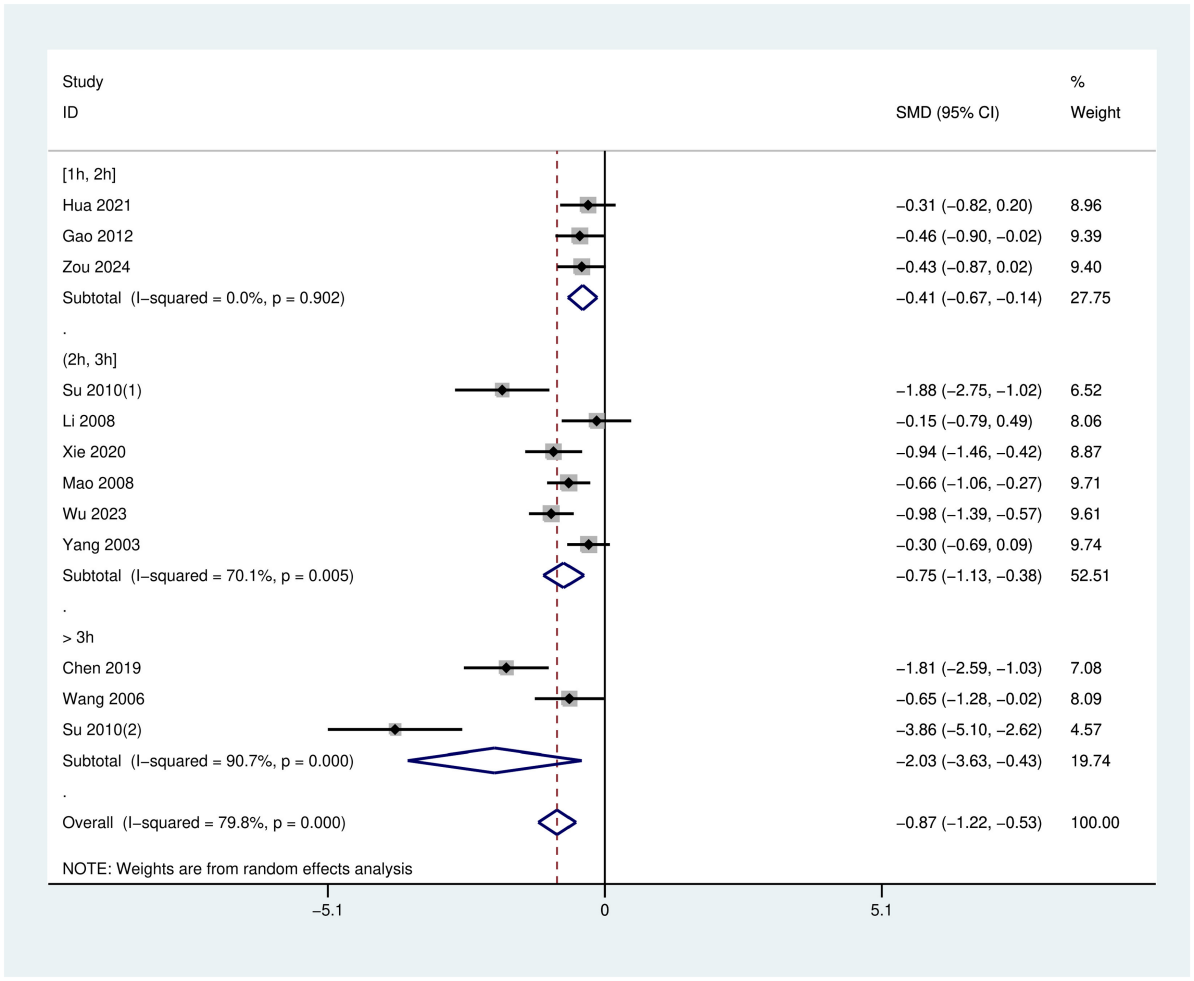
Forest plot for subgroup analysis of weekly training time in Tai Chi.

Between-group comparisons revealed that the intervention effect was optimal when the weekly training time was >3 h. In contrast, the intervention effect was poorest when the weekly training time was ≥1 h and ≤2 h. The effect was intermediate when the intervention weekly training time was >2 h and ≤3 h, as shown in the Figure 6.

### Sensitivity analysis

**Figure 7:**
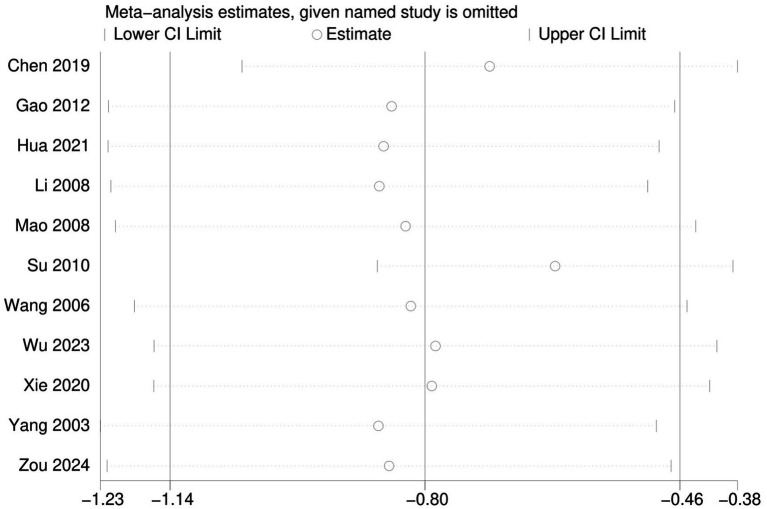
Sensitivity analysis of depression indicators.

Sensitivity analysis was conducted on the depressive symptom score. The results indicated that the combined effect sizes remained within the 95% confidence interval even after the exclusion of each individual study. This finding suggests that the results of this study are robust and reliable, as shown in Figure 7.

### Publication bias analysis

**Figure 8:**
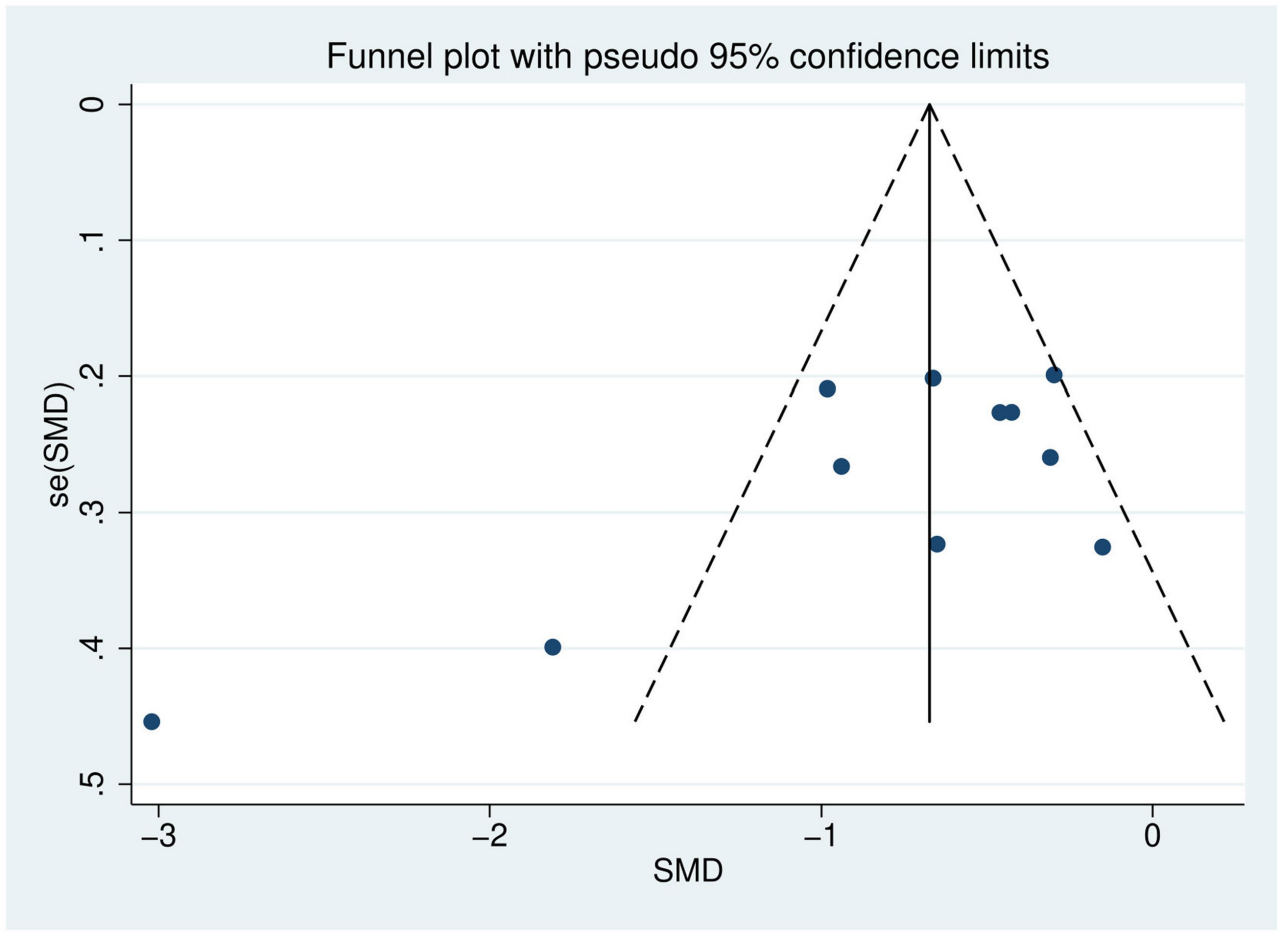
Depression index funnel plot.

Funnel plots, Egger’s test and Begg’s test were used to assess publication bias. The funnel plot showed a slight asymmetry (Figure 8). In addition, the results of Egger’s test (*p* = 0.042) were less than 0.05, and the results of Begg’s test (*p* = 0.213) were greater than 0.05, suggesting that there may be publication bias (Supplementary Figures 4–6). To assess the effect of publication bias on the results, we used the trim-and-fill method (Supplementary Figures 7, 8). The combined effect size before correction was SMD = −0.80, 95% CI (−1.14, −0.46), *I*^2^ = 79.30%. The trim-and-fill method detected funnel plot asymmetry and filled 2 hypothetical studies. The corrected combined effect size was SMD = −0.94, 95% CI (−1.29, −0.60), *I*^2^ = 83.80%. Despite the presence of publication bias, the corrected results support the original conclusions.

## Discussion

In recent years, Tai Chi, as a traditional Chinese medicine exercise therapy, has been increasingly employed as an auxiliary treatment for depression (28). However, the effect of Tai Chi alone on improving depressive mood in young individuals aged 15–24 years, as well as its optimal dosage, remains unclear. Recent studies have suggested that Tai Chi may alleviate depressive symptoms through multiple physiological mechanisms. These include modulation of the hypothalamic–pituitary–adrenal axis, leading to reduced cortisol secretion and stress response (29), as well as increased levels of brain-derived neurotrophic factor, which supports neuroplasticity and emotional regulation (30). Moreover, the meditative and rhythmic nature of Tai Chi practice has been associated with improvements in autonomic nervous system balance and reductions in inflammatory and oxidative stress markers (31–33). Although these mechanisms were not directly assessed in our study, they are supported by a growing body of evidence.

The study highlights the therapeutic potential of Tai Chi in mitigating depressive mood among young individuals aged 15–24 years. Based on previous studies (34), engaging in Tai Chi is suggested to increase the secretion of endorphins, which are commonly referred to as the body’s natural “feel-good” chemicals and may contribute to mood elevation. Additionally, the practice’s requirement for concentration and the coordination of slow movements serves as a mental distraction from negative thoughts (35). This dual effect of biochemical and psychological benefits makes Tai Chi a promising intervention for managing depression.

For young individuals aged 15–24 years with depressive mood, school-based Tai Chi programs should provide at least three standardized 60-min sessions weekly to meet the optimal dosage of >3 h/week identified in our subgroup analysis. Conducting the program for 12 weeks achieves significant symptom reduction. Implementation requires careful scheduling to ensure consistent delivery while accommodating academic timetables. We recommend formal partnerships with regional Tai Chi associations to provide certified instructors. Clinical oversight through campus mental health services is crucial for participants with severe symptoms or suicide risk.

### Interpretation of heterogeneity patterns

The meta-regression incorporating six clinically relevant covariates (gender distribution, intervention type, intervention period, weekly training time, exercise intensity, and assessment tools) did not identify significant moderators of heterogeneity. The absence of statistical significance (all covariates *p* > 0.05) may reflect limited statistical power due to the modest number of studies (*n* = 11) combined with substantial between-study variance (*τ*^2^ = 0.5586). The persistent high residual heterogeneity (*I*^2^_res_ = 84.12%) suggests contributions from unmeasured factors such as baseline depression severity, comorbid conditions, or methodological variations in intervention delivery protocols. Future trials should prioritize standardized reporting of participant characteristics and implementation parameters to facilitate more conclusive heterogeneity exploration.

### Comparison of this study with other studies

Previous studies (17, 36) have often used Tai Chi in integration with other treatments to improve depressed mood in young individuals. The present study, on the other hand, was targeted to examine the improvement of Tai Chi alone intervention on depressed mood in young individuals. The results of this study indicate that Tai Chi training can effectively reduce the depression scale scores in young individuals with depressive mood. Interventions lasting fewer than 12 weeks failed to produce a significant effect, likely due to insufficient time for movement proficiency development. However, a significant effect emerged after 12 weeks of intervention (SMD = −1.11). Interventions exceeding 12 weeks were effective but suboptimal, limited by individual fatigue. Regarding practice duration, practice ≤2 h/week was ineffective, 2–3 h/week was moderately effective, and only >3 h/week yielded the greatest clinical benefit (SMD = −2.03). These findings demonstrate a clear dose–response relationship. Notably, training for 12 weeks with a duration exceeding 3 h per week, is associated with a greater improvement in these scores. Zeng et al. reported a 24 weeks Tai Chi program which significantly alleviated depressive symptoms in middle-aged and older adults, whose effect was better pronounced when the total duration of the intervention exceeded 40 h (13). Chang et al. (14) found that older women who participated in at least 12 weeks of Tai Chi exercise, each session lasting 1 h, experienced significant improvements in depression. In contrast to prior researches, the intervention period and weekly training time varied among the studies included in our analysis. These discrepancies in intervention protocols may result in differing outcomes. This study aimed to identify the most effective intervention protocols for young individuals aged 15–24 years by assessing their depression scale scores. Moreover, while prior researches primarily focused on middle-aged and older populations, our findings extend this research to include young individuals aged 15–24 years, suggesting that the therapeutic effects of Tai Chi are not confined to middle-aged and older populations.

### Limitations and suggestions for future research

Several limitations need to be recognized in this study. First, the 24-style Tai Chi, developed by the Chinese Sports Committee in 1956, is a simplified version of Yang-style Tai Chi consisting of 24 standardized movements. It features slow, continuous, and structured motions, making it easy to learn and suitable for large-scale health interventions. It has been widely applied in psychological health studies (37). The participants included in the current study were primarily used the 24-style Tai Chi, which means that in the future, more studies that include different styles of Tai Chi need to be completed. Second, the differences in the number of weeks of Tai Chi intervention, weekly training time, and depressive state rating scales resulted in a relatively high degree of heterogeneity among the studies. Third, although we conducted a comprehensive search of both English and Chinese databases, the included RCTs were published primarily in Chinese. The exclusion of studies in other languages may limit the generalizability of our findings and should be addressed in future research. Fourth, all included trials assessed depressive symptoms only at baseline and immediately after the Tai Chi intervention, without any post-intervention follow-up. As a result, the long-term sustainability of Tai Chi’s antidepressant effects in young individuals aged 15–24 years remains uncertain. Future RCTs should incorporate follow-up assessments to evaluate whether the observed benefits persist in the absence of supervised practice. Fifth, only two studies described adequate allocation concealment methods, potentially introducing selection bias. Furthermore, blinding of participants and personnel was universally unattainable due to the nature of exercise-based interventions, which may introduce performance bias through placebo effects. Sixth, all included trials were exclusively conducted within China, which constrains generalizability to other cultural contexts. We explicitly acknowledge this geographical limitation and emphasize the need for multinational replications. Seventh, although control group participants were instructed to avoid structured mind–body interventions, other potentially influential activities including social interactions and dietary modifications were not systematically monitored, representing a significant source of potential confounding.

## Conclusion

Our study highlights the potential of Tai Chi as an effective non-pharmacologic intervention for managing depressive mood in young individuals aged 15–24 years. The benefits observed were especially pronounced during longer and more intensive Tai Chi training sessions. Future research should build on these findings to optimize Tai Chi exercise programs and promote their widespread implementation in the context of youth mental health.

## Data Availability

The original contributions presented in the study are included in the article/Supplementary material, further inquiries can be directed to the corresponding author.
