# Supplementary figures and images for: The effectiveness of Tai Chi in improving depressive mood among young individuals aged 15–24 years: a systematic review and meta-analysis
Source: Front Public Health. 2025 Sep 3;13:1517350. doi: 10.3389/fpubh.2025.1517350 (PMC12440746; doi:10.3389/fpubh.2025.1517350)

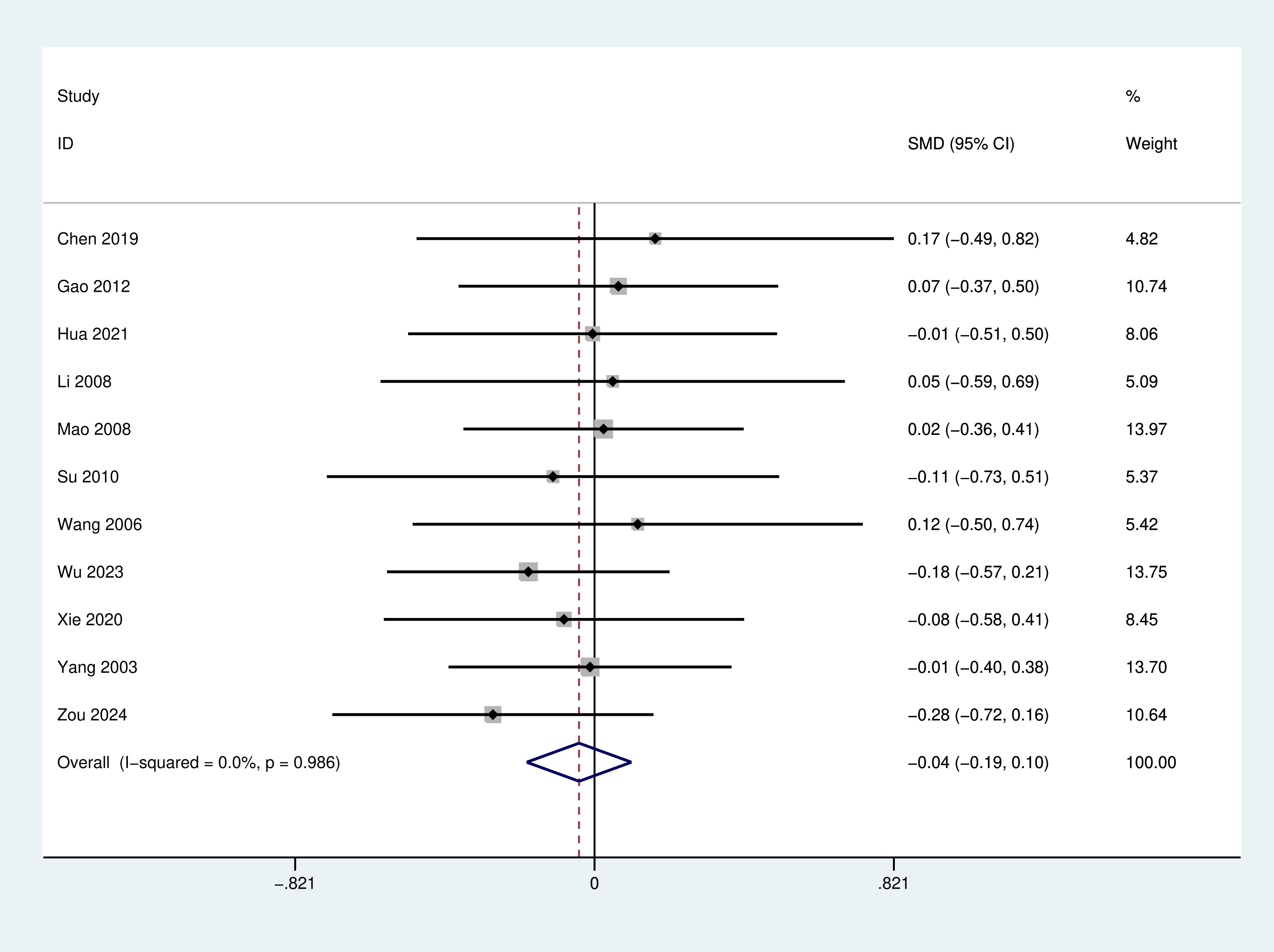

Supplement: Supplementary file 2 [file Data_Sheet_2.ZIP › Supplementary Figure/Supplementary Figure 1.jpg]

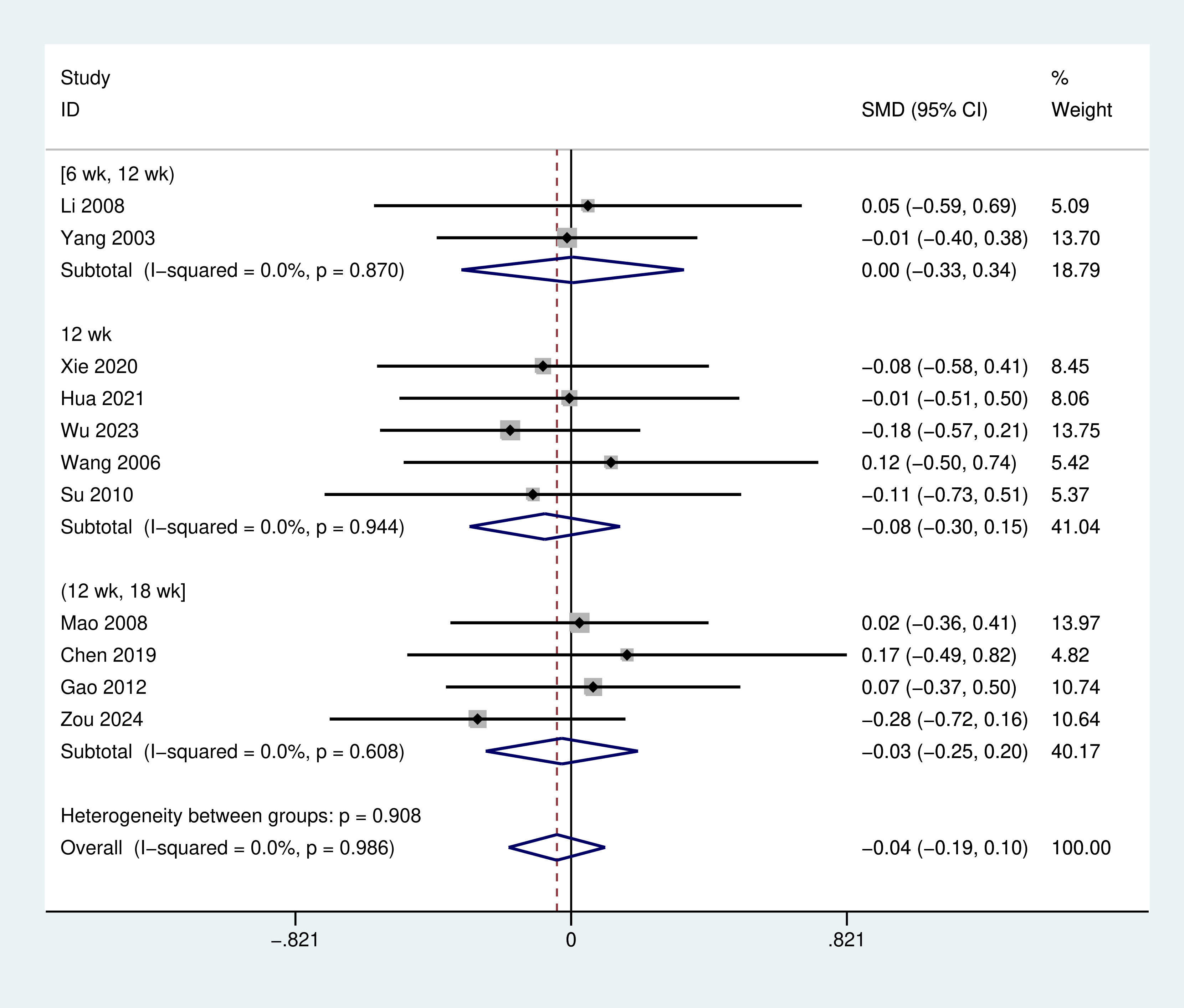

Supplement: Supplementary file 2 [file Data_Sheet_2.ZIP › Supplementary Figure/Supplementary Figure 2.jpg]

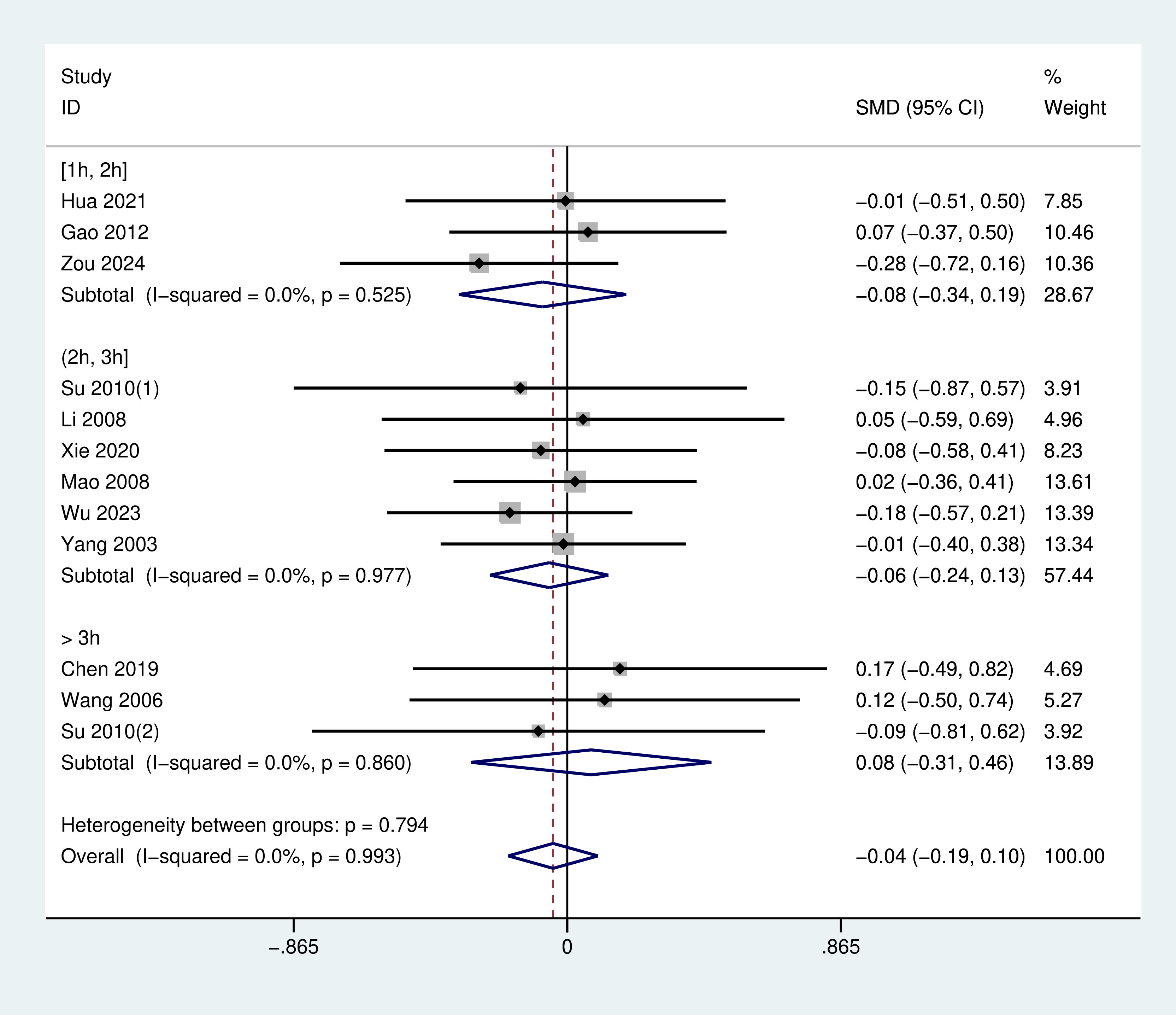

Supplement: Supplementary file 2 [file Data_Sheet_2.ZIP › Supplementary Figure/Supplementary Figure 3.jpg]

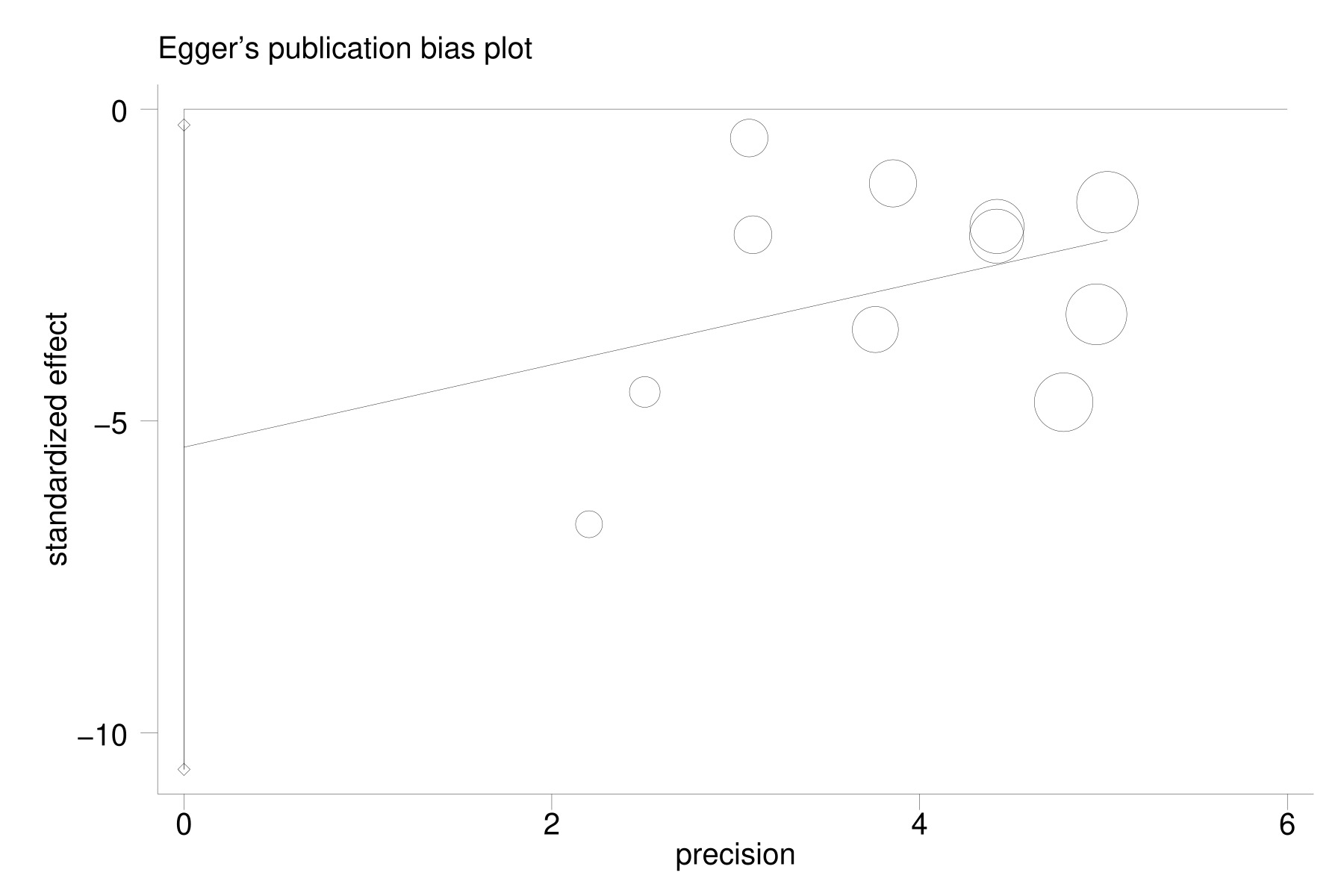

Supplement: Supplementary file 2 [file Data_Sheet_2.ZIP › Supplementary Figure/Supplementary Figure 4.jpeg]

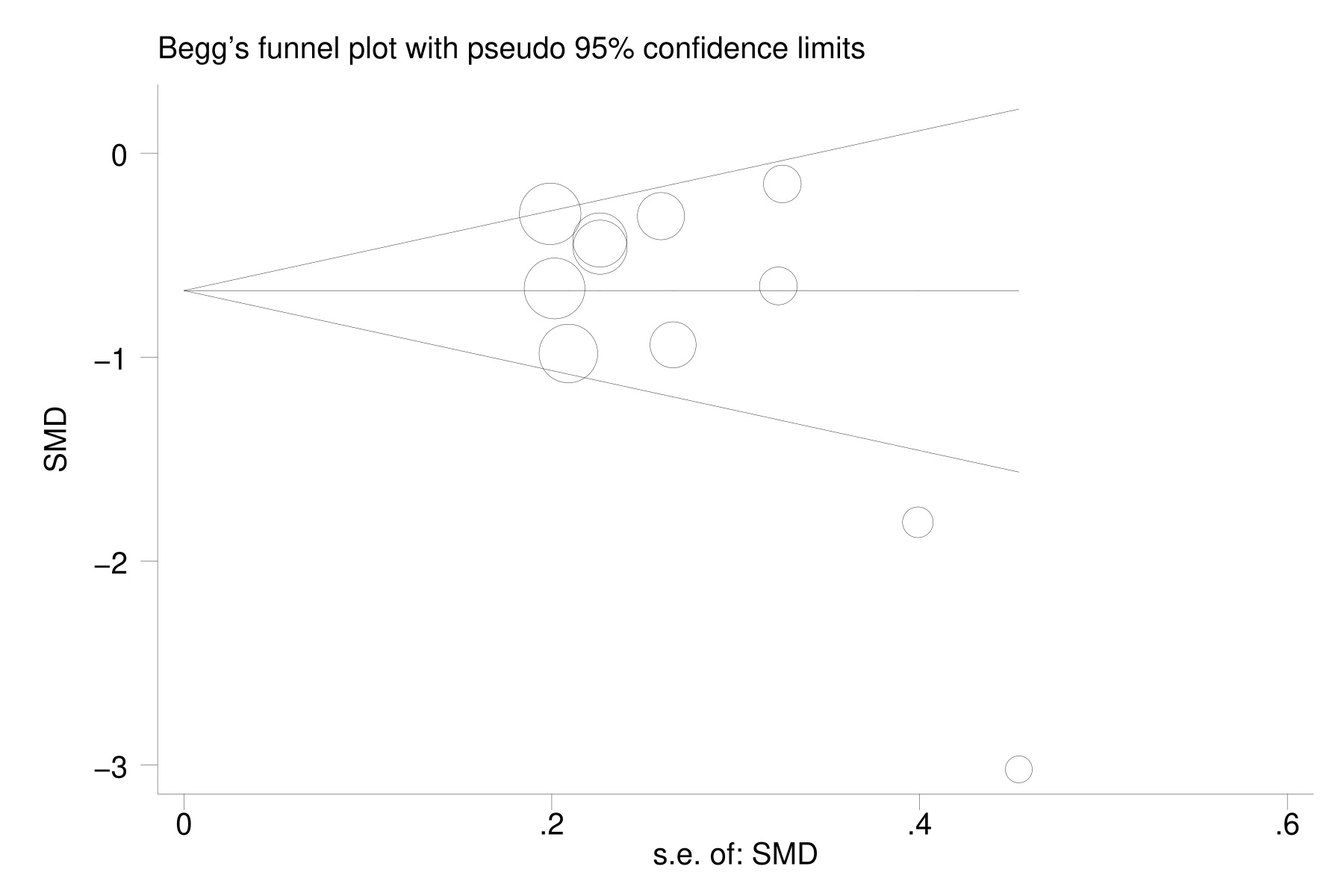

Supplement: Supplementary file 2 [file Data_Sheet_2.ZIP › Supplementary Figure/Supplementary Figure 5.jpeg]

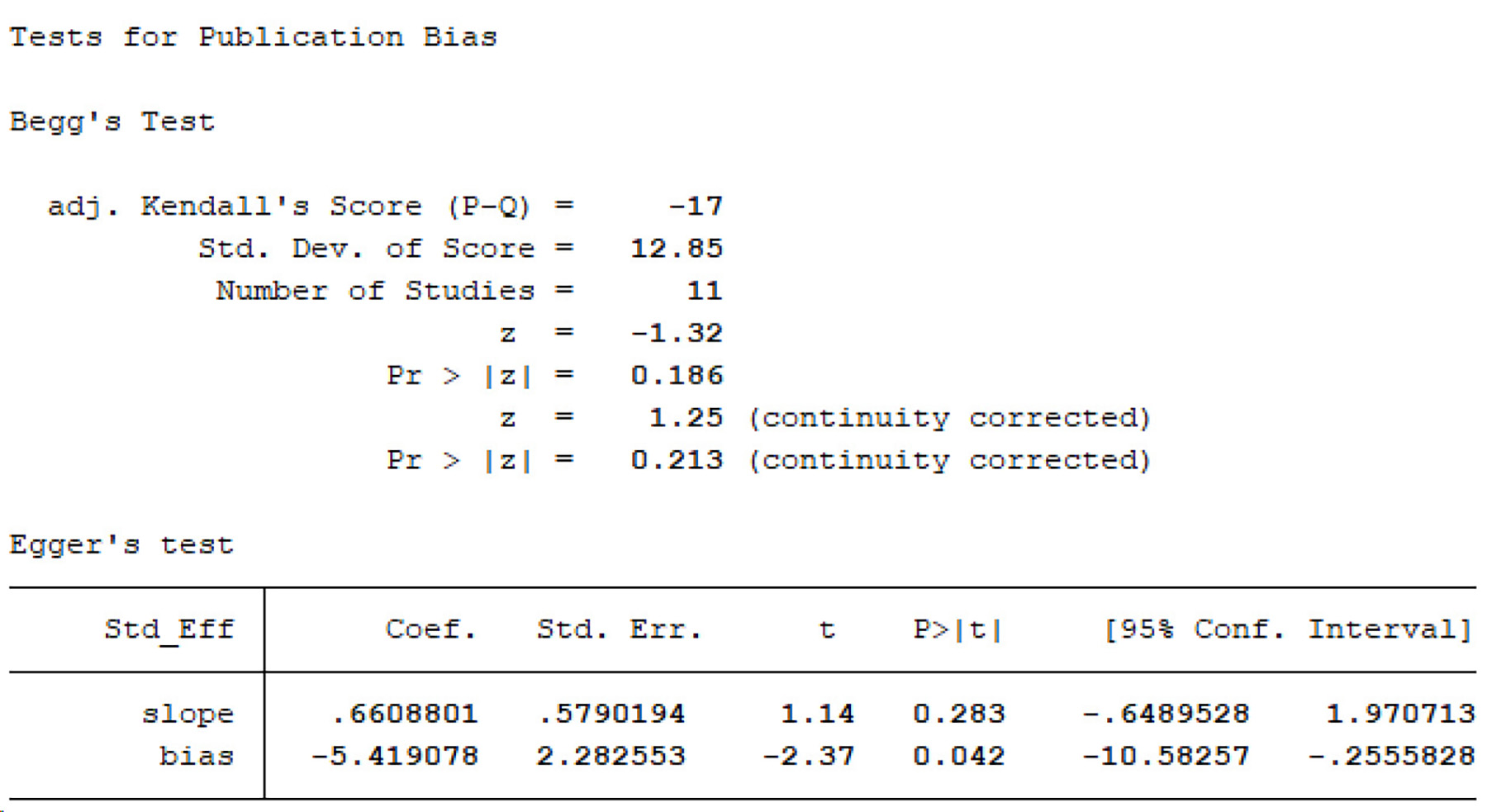

Supplement: Supplementary file 2 [file Data_Sheet_2.ZIP › Supplementary Figure/Supplementary Figure 6.jpg]

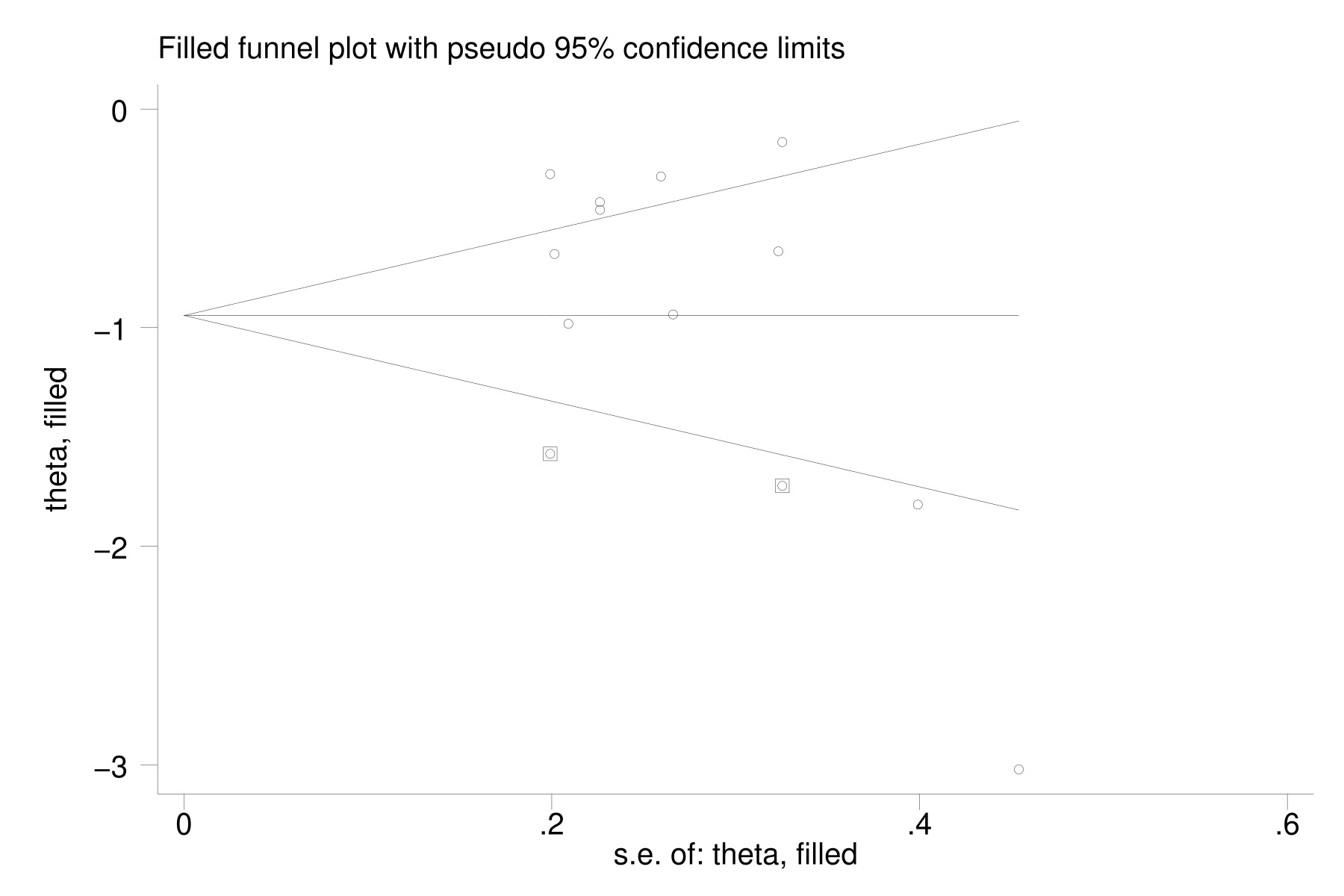

Supplement: Supplementary file 2 [file Data_Sheet_2.ZIP › Supplementary Figure/Supplementary Figure 7.jpeg]

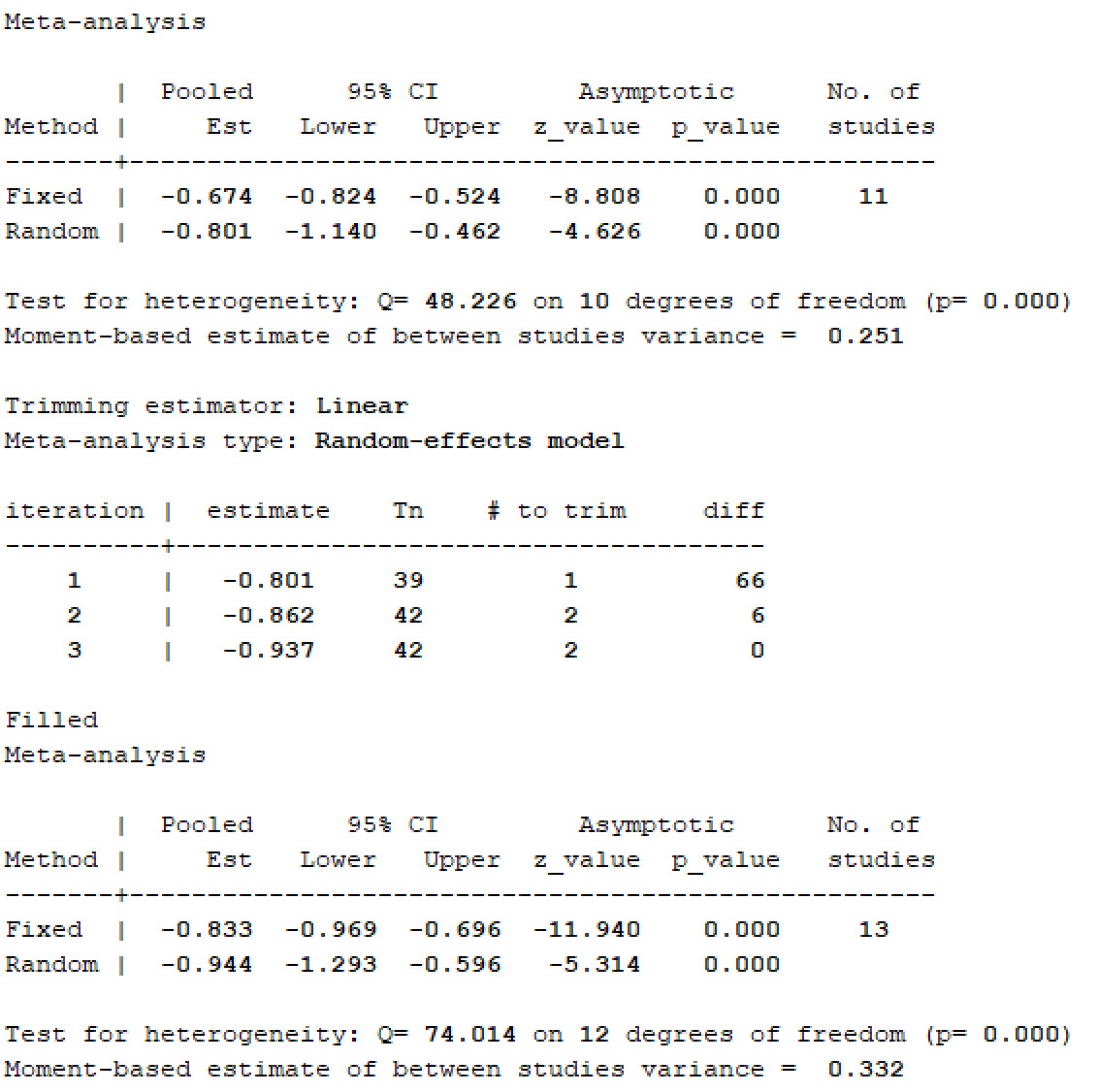

Supplement: Supplementary file 2 [file Data_Sheet_2.ZIP › Supplementary Figure/Supplementary Figure 8.jpeg]
